# Profound Sustained Hypotension Following Renal Denervation: A Dramatic Success?

**DOI:** 10.5935/abc.20150100

**Published:** 2015-08

**Authors:** Ganiga Srinivasaiah Sridhar, Timothy Watson, Chee Kok Han, Wan Azman Wan Ahmad

**Affiliations:** 1 Departamento de Cardiologia - Universidade Malaya Medical Center - Kuala Lumpur Malaysia; 2 Departamento de Medicina - Universidade de Auckland - Auckland, New Zealand

**Keywords:** Hypertension, Sympatectomy, Medication Therapy, Management, Hypotension

## Introduction

A 67-year-old woman with drug-refractory essential hypertension was admitted for renal sympathetic denervation (RDN). The secondary causes of hypertension were fully investigated in this patient. A 24-h ambulatory blood pressure (BP) monitor documented a mean daytime BP of 172/101 mmHg, a mean nighttime BP of 151/84 mmHg, and an overall mean BP of 167/97 mmHg despite compliance with metoprolol (50 mg twice daily), amlodipine (10 mg once daily), lisinopril (20 mg once daily), prazosin (2 mg thrice daily), and hydrochlorothiazide (50 mg once daily).

**Figure 1:**
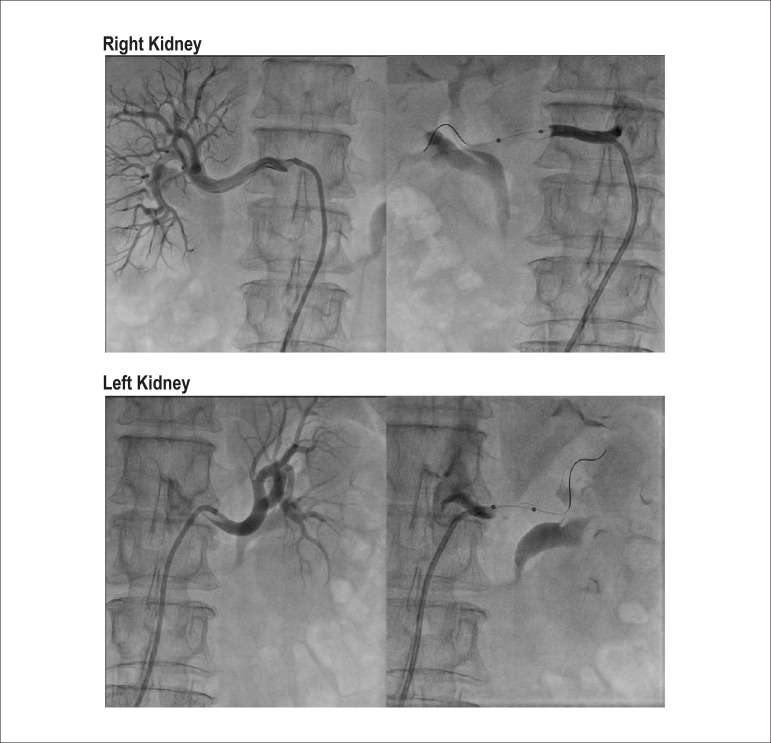
Renal Denervation Procedure. Selective angiography performed with a 7-F Renal Double Curve guide catheter in the left anterior oblique 10° projection. Note that a single renal artery supplies each kidney and that the caliber and length of the main renal artery prior to bifurcation is ideally suited to denervation.

The patient was fasted for 4 h. Her usual antihypertensive drug therapy was continued. After the administration of 5,000 international units of heparin and 100 *μ*g of fentanyl, a 7-F Renal Double Curve guide catheter (Cordis Corporation, Fremont, CA ,USA) was inserted into the right renal artery (no accessory vessel). A 0.014-in Runthrough floppy guide wire (Terumo Medical Corporation, Somerset, NJ, USA) was advanced into place. A 6-mm ONESHOT^TM^ (Covidien, Mansfield, MA, USA) irrigated RDN balloon was advanced into place (Figure 1), and a single ablation was performed. The procedure was then repeated on the other side. The patient remained hemodynamically stable throughout and at the completion of the procedure with a BP of 150/80 mmHg. Hemostasis was achieved with the Perclose ProGlide Suture-Mediated Closure System (Abbott Vascular, Santa Clara, CA, USA), and the patient was then returned to our ward for monitoring.

One hour later, the patient complained of dizziness and blurring of vision. Her Glasgow Coma Scale score remained 15 with preserved mentation. She was not in pain. Her pulse rate was 87 bpm, and her BP was 77/38 mmHg. However, she appeared well perfused and was clinically euvolemic. There was no evidence of a groin hematoma, and her abdomen was soft. A 12-lead electrocardiogram showed no change, and a transthoracic echocardiogram showed normal left ventricular function. Her hemoglobin level was similar to baseline, and her arterial blood gas levels, including lactate, were unremarkable.

She was given intravenous dopamine that was titrated to response. At 10 *μ*g/kg/min, her BP rose to 120/70 mmHg, and her symptoms resolved entirely. Over the subsequent 48 h, she was extremely sensitive to reductions in the dose of dopamine and exhibited a markedly fluctuating BP. However, by 72 h, the dopamine had been carefully weaned and discontinued. She was discharged home after she remained stable for a further 24 h. At a 3-month review, she remains well with a mean daytime office BP of 124/72 mmHg while on amlodipine (5 mg once daily).

## Discussion

Around 12% of patients with essential hypertension who are considered resistant to conventional therapy have persistently elevated BP despite the use of three or more pharmacological agents^1,2^. In such instances, abnormal renal excretory function, which is largely influenced by renal sympathetic nerve activity, may have a central role^3^. Catheter-based RDN, which is a modern incarnation of a historically effective treatment, has recently emerged as a novel therapeutic strategy. Proof of concept and subsequent randomized (unblinded) data that were collected while using the Symplicity**®** catheter (Medtronic, Inc., Minneapolis, MN, USA) have demonstrated reductions in office BP of 20/10, 24/11, 25/11, and 23/11 mmHg at 1, 3, 6, and 12 months, respectively, in a group of patients taking an average of five antihypertensive drugs^4,5^. Such early exciting reports have stimulated the development of numerous other similar devices, including the ONESHOT^TM^ catheter that was used in this case^6^.

In some cases, early BP reductions have been reported following RDN. However, in other cases, the response is not always immediate, or it can take several months to appear. Additionally, RDN is associated with a failure rate of 10%-30%, and the only predictor of response in early studies is the magnitude of the systolic BP elevation at baseline^7^. Explanations for this broad variability in outcome are uncertain, except that raw BP measurements may lack the sensitivity required for them to be considered a true measure of successful RDN^8^. This may in part explain the lackluster performance of RDN in the Symplicity-3 trial, in which RDN failed to demonstrate superiority over conventional treatments when compared to a sham-control procedure^9^. However, despite this, the use of RDN does seem to significantly reduce renal norepinephrine spillover^10^. Therefore, it is possible that those patients who exhibit more sympathetic over-activity may experience a greater degree of BP reduction with RDN. Because sympathetic over-activity is not routinely measured in clinical practice, this remains speculative.

Nonetheless, the role of RDN in the treatment of resistant hypertension remains uncertain, but the sustained and impressive BP reductions that were observed in this and other cases should encourage further research to improve the understanding of the mechanisms through which hypertension is mediated and to identify those patients who are likely to achieve the most dramatic responses with RDN.
